# Supplementary material for: The interactome of intact mitochondria by cross-linking mass spectrometry provides evidence for coexisting respiratory supercomplexes
Source: Mol Cell Proteomics. 2017 Dec 8;17(2):216–32. doi: 10.1074/mcp.RA117.000470 (PMC5795388; doi:10.1074/mcp.RA117.000470)
Supplement: Supplemental Data [file supp_RA117.000470_133922_0_supp_23978_fzffwf.zip › spectra_annotation/mito_DR_spectra_annotation/60-1-16-1-21-1.pdf]

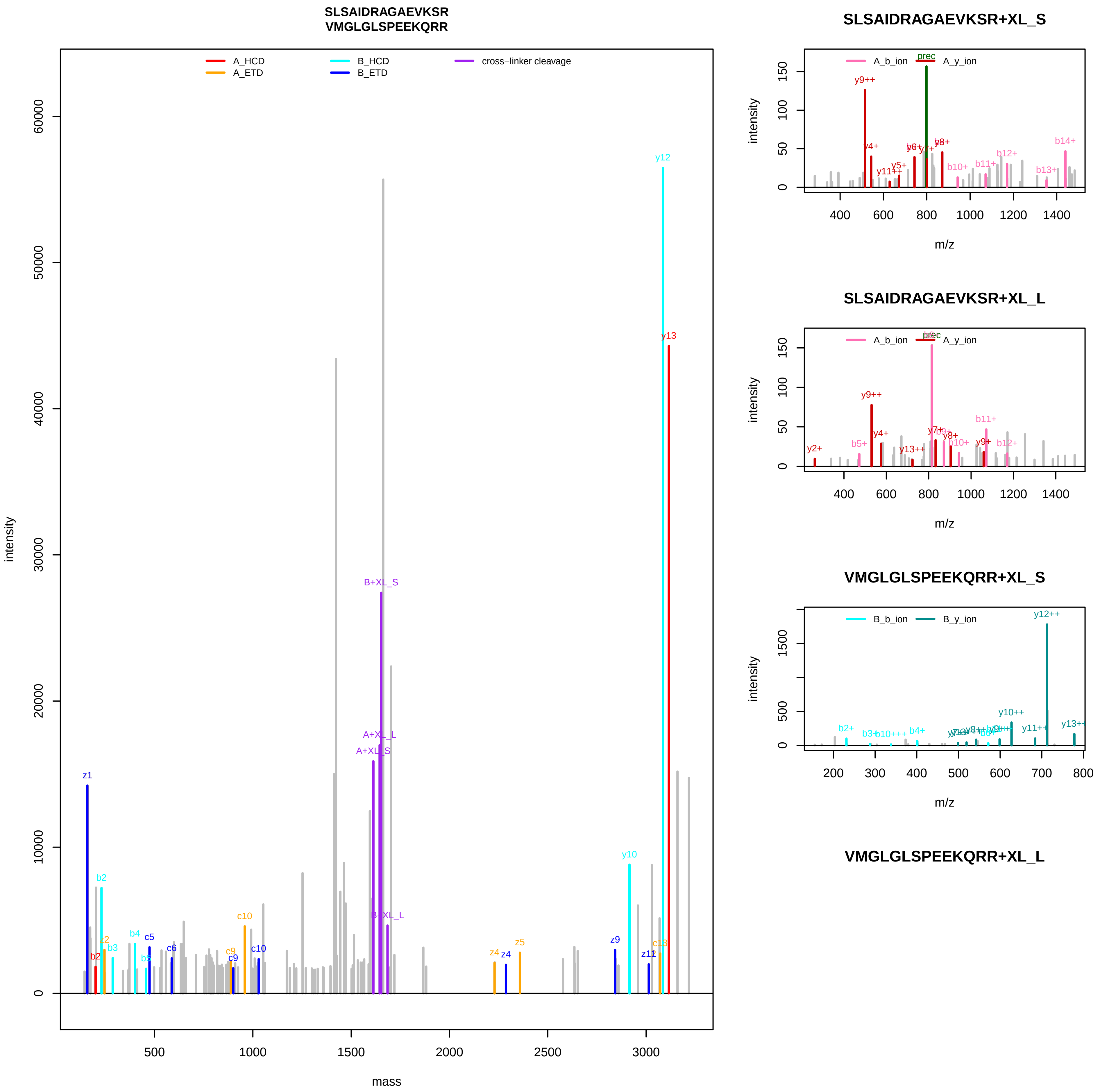

SLSAIDRAGAEVKSR
VMGLGLSPEEKQRR
A_HCD
A_ETD
B_HCD
B_ETD
cross−linker cleavage
intensity
mass
0
10000
20000
30000
40000
50000
60000
500
1000
1500
2000
2500
3000
z1
b2
b2
z2
b3
b4
c5
b5
c6
c9
c9
c10
c10
B+XL_S
A+XL_L
A+XL_S
B+XL_L
z4
z4
z5
z9
z11
c13
y10
y12
y13
SLSAIDRAGAEVKSR+XL_S
A_b_ion
A_y_ion
prec
y9++
y4+
y11++
y5+
y6+
y7+
y8+
b10+
b11+
b12+
b13+
b14+
intensity
m/z
SLSAIDRAGAEVKSR+XL_L
y2+
b5+
y9++
y4+
y13++
y7+
y8+
b10+
y9+
b11+
b12+
VMGLGLSPEEKQRR+XL_S
B_b_ion
B_y_ion
b2+
b4+
y10++
y11++
y12++
VMGLGLSPEEKQRR+XL_L
